# Planar Microwave Sensor for Theranostic Therapy of Organic Tissue Based on Oval Split Ring Resonators

**DOI:** 10.3390/s16091450

**Published:** 2016-09-08

**Authors:** Carolin Reimann, Margarita Puentes, Matthias Maasch, Frank Hübner, Babak Bazrafshan, Thomas J. Vogl, Christian Damm, Rolf Jakoby

**Affiliations:** 1 Institute for Microwave Engineering and Photonics, Technische Universität Darmstadt, Merckstr. 25, Darmstadt 64283, Germany; puentes@imp.tu-darmstadt.de (M.P.); jakoby@imp.tu-darmstadt.de (R.J.); 2 Terahertz Sensors Group, Technische Universität Darmstadt, Merckstr. 25, Darmstadt 64283, Germany; maasch@imp.tu-darmstadt.de (M.M.); damm@imp.tu-darmstadt.de (C.D.); 3 Institute of Diagnostic and Interventional Radiology, Goethe University, Theodor-Stern-Kai 7, Frankfurt am Main 60590, Germany; Frank.Huebner@kgu.de (F.H.); Babak.Bazrafshan@kgu.de (B.B.); Thomas.Vogl@kgu.de (T.J.V.)

**Keywords:** theranostics, oval split ring resonator, microwave ablation

## Abstract

Microwave sensors in medical environments play a significant role due to the contact-less and non-invasive sensing mechanism to determine dielectric properties of tissue. In this work, a theranostic sensor based on Split Ring Resonators (SRRs) is presented that provides two operation modes to detect and treat tumor cells, exemplary in the liver. For the detection mode, resonance frequency changes due to abnormalities are evaluated, and in the treatment mode, microwave ablation is performed. The planar sensor structure can be integrated into a needle like a surgery tool that evokes challenges concerning size limitations and biocompatibility. To meet the size requirements and provide a reasonable operating frequency, properties of oval shaped SRRs are investigated. By elongating the radius of the SRR in one direction, the resonance frequency can be decreased significantly compared to circular SRR by a factor of two below 12 GHz. In order to validate the detection and treatment characteristics of the sensor, full wave simulations and measurements are examined. Clear resonance shifts are detected for loading the sensor structures with phantoms mimicking healthy and malignant tissue. For treatment mode evaluation, ex vivo beef liver tissue was ablated leading to a lesion zone 1.2 cm × 1 cm × 0.3 cm with a three minute exposure of maximum 2.1 W.

## 1. Introduction

The future of medicine is going in the direction of personalized treatments for each individual patient. A promising approach is the use of theranostic devices or systems that provide a combination of diagnosis and therapy. The objective of theranostics is to apply a personalized therapy plan for each patient to improve the individual treatment success. For this purpose, microwave devices are well suited. Especially in cancer therapy, microwave technology could prove very useful in diagnostics and for treatment. The main advantages of microwaves are the contact-less and non-destructive sensing mechanism as well as the good electromagnetic wave penetration properties into human tissue.

Nowadays, imaging techniques are widely used for tumor detection with the most established systems being computed tomography (CT) and magnetic resonance imaging (MRI). However, CT brings the disadvantage of ionizing radiation to the patient. MRI solves this problem by applying a gradient in a static magnetic field. However, this imaging process is very time consuming and not suitable for claustrophobic and adipose people. An emerging technique for tumor detection is microwave imaging where dielectric properties of tissue are evaluated in order to detect malignancies that can be seen as a complementary approach to standard imaging techniques [1]. Generally, malignant and non-malignant tissue can be distinguished by microwaves due to different dielectric properties. These are strongly dependent on the type of tissue of the tumor.

Regarding cancer treatment, current techniques are based on radiotherapy, chemotherapy and open surgery. However, thermal ablation presents an alternative treatment option with increasing significance [2]. The provided temperature rise at a specific location is sufficient to eradicate tumor cells with the advantages of a less invasive therapy compared to open surgery. Additionally, thermal ablation can be performed very precisely so that difficult accessible tumors and tumors located deep inside the organs can be treated with a lower risk to damage important blood vessels. All in all, thermal therapy leads to a shorter recovery time for the patient and less risk for complications. During the ablation therapy, the surgeon targets the tumor mostly with the help of continuous CT imaging.

In this work, a theranostic microwave device is proposed that improves current ablation therapy systems by adding a sensing mechanism to the applicator to detect malignant cells. With this theranostic approach, the accurate localization and precise treatment at the right timing and proper dose becomes possible. Furthermore, monitoring of the treatment progress is enabled. The proposed applicator is aimed to be integrated in a minimally invasive surgery tool that leads to a strict size limitation for such a theranostic structure. Therefore, the key elements of the sensor are split ring resonators (SRRs) with the feature of a physically small size that are coupled to a coplanar waveguide. The SRRs are excited in two different modes: the detection and treatment mode. For the detection mode, a frequency sweep around the resonance of the SRR with low input power is applied and relative changes of resonance frequency are evaluated in order to extract permittivity changes due to tumor cells. In the treatment mode, the input power is increased at the specific resonance frequency resulting in the generation of heat at the surrounding tissue of the operation tool.

The detection of relatively small variations of permittivity values caused by abnormalities is a challenging factor for the sensor design process. Therefore, a comprehensive study of SRRs as sensing particles that are integrated in a minimally invasive surgery applicator is examined. Moreover, the thermal characteristics and size of the ablation zone are analyzed in Section 2. Section 3 introduces the fabricated prototype and experimental setup for the validation of the detection mode and treatment mode, respectively. In Section 4, the corresponding measurement results are shown that are further discussed in Section 5. Finally, conclusions are given in Section 6.

## 2. Theoretical Considerations

The proposed theranostic microwave sensor is designed for use as a needle-like surgery tool for minimally invasive operations. From that field of application, a strict size limitation can be derived that is 2 mm × 2 mm × 200 mm. Consequently, the operating frequency of such a small device is relatively high. Although SRRs as subwavelength resonators provide a small physical size of about one-tenth of the free space wavelength, the sensor design with pairs of circular SRR coupled to a coplanar waveguide for the given size limitation would lead to the operating frequency range above 30 GHz [3]. The cost and effort to perform high power thermal ablation treatment in this frequency range are not practicable for future scenarios as a surgical tool in a clinical environment. Hence, the resonance frequency of the SRRs is a critical design parameter that leads to a trade-off between the physical size of the device and the system complexity for the future application. The prospective compromise is to design a sensor tool that works in the X-band between 8 GHz and 12 GHz. A further advantage of decreasing the resonance frequency below 12 GHz is evident by considering the penetration depth of an electromagnetic field into tissue. Generally, the relative permittivity of biological material has a complex form that can be written as $\varepsilon_{r}=\varepsilon_{r}^{\prime }-j\varepsilon_{r}^{\prime \prime }$, while the permeability is close to that of free space. The penetration depth is defined as the depth where the electric field density has decreased to 1/e of the initial value at the surface of the applicator. Assuming that the condition $\varepsilon_{r}^{\prime \prime }\gg \varepsilon_{r}^{\prime }$ is valid for tissue due to ion mobility [4], the penetration depth $\delta_{p}$ can be approximated by

(1) $$\delta_{p}\approx \frac{1}{\omega }\sqrt{\frac{2}{\mu_{0}\varepsilon_{0}\varepsilon_{r}^{\prime \prime }}},$$

where $\mu_{0}$ and $\varepsilon_{0}$ describe the permeability and permittivity of free space, respectively. According to Equation (1), a larger penetration depth of microwave energy into tissue is achieved for lower frequencies. All in all, the objective to develop a device with decreased operating frequency that fulfills the given size requirements is achieved by investigating oval shaped SRR structures as sensing elements.

### 2.1. Oval Split Ring Resonators

**Figure 1:**
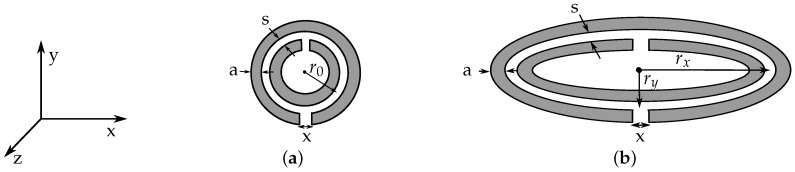
Drawings of circular (**a**) and oval Split Ring Resonator (SRR) (**b**) with corresponding dimensions.

The general structure of the sensor device is based on pairs of oval SRRs coupled to a coplanar waveguide as investigated in [5]. In Figure 1, the schematic drawings of a circular and an oval SRR with corresponding dimensions are shown. From the property of SRRs providing a small electrical size, a quasi-static equivalent circuit model can be applied that corresponds to an LC resonant circuit. When a SRR is excited by a time varying magnetic field along the *z*-axis, a current is induced along the rings. The resonance frequency of circular SRR can be calculated as [6]

(2) $$f_{0}=\frac{1}{2\pi }\;\cdot \;\sqrt{\frac{1}{L_{\mathrm{SRR}}C_{\mathrm{SRR}}}},$$

where $L_{\mathrm{SRR}}$ is the total inductance of the SRR, and $C_{\mathrm{SRR}}$ describes the total capacitance that is composed by the capacitance between the rings and the gap capacitance. A detailed electromagnetic analysis in [7] leads to the consideration that the total capacitance $C_{\mathrm{SRR}}$ is determined by two series capacitances of the two halves of the SRR. For a circular SRR, the total capacitance yields

(3) $$C_{\mathrm{SRR}}=\frac{\pi r_{0}C_{p.u.l.}}{2},$$

where $C_{p.u.l.}$ is the per unit length capacitance between the rings and $r_{0}$ is the average radius of both rings of the SRR according to Figure 1a. The inductance $L_{\mathrm{SRR}}$ can be computed by the inductance of a single ring with average radius of both concentric split rings $r_{0}$ and circular cross section with diameter equal to the width *a* of the original rings. The self inductance of a ring with a circular cross section can be approximated by [8]

(4) $$\begin{array}{c} L_{\mathrm{SRR}}=\mu_{0}r_{0}\left(\ln\frac{2\;\cdot \;8r_{0}}{a}-1.75\right). \end{array}$$

For the elliptical shaped SRR, both the inductance and capacitance of SRR are modified due to the varied circumference of an ellipse $U_{\mathrm{Ellipse}}$ compared to a circle that is given by [9]

(5) $$U_{\mathrm{Ellipse}}=4r_{x}E\left(e,\frac{\pi }{2}\right),$$

with *E* being the complete elliptic integral of second kind

(6) $$E\left(e,\frac{\pi }{2}\right)=\int_{0}^{\pi /2}\sqrt{1-e^{2}\sin^{2}\varphi }\;d\varphi .$$

The integral is a function of the eccentricity $e=\sqrt{r_{x}^{2}-r_{y}^{2}/r_{x}}$ of the ellipse and can be solved numerically. Here, the radius $r_{x}$ describes the semi-major axis and $r_{y}$ the semi-minor axis of the ellipse. By including this geometrical relation into Equations (3) and (4), the following adjusted equations for the inductance $L_{\mathrm{oval}\;\mathrm{SRR}}$ and capacitance $C_{\mathrm{oval}\;\mathrm{SRR}}$ can be formulated and inserted in Equation (2) to compute the resonance frequency of an oval SRR:

(7) $$\begin{array}{c} L_{\mathrm{oval}\;\mathrm{SRR}}=\frac{2}{\pi }\mu_{0}r_{x}E\left(e\right)\left(\ln\frac{32r_{x}E\left(e\right)}{\pi a}-1.75\right), \end{array}$$

(8) $$\begin{array}{c} C_{\mathrm{oval}\;\mathrm{SRR}}=2r_{x}E\left(e\right)\;\cdot \;C_{p.u.l.}. \end{array}$$

**Figure 2:**
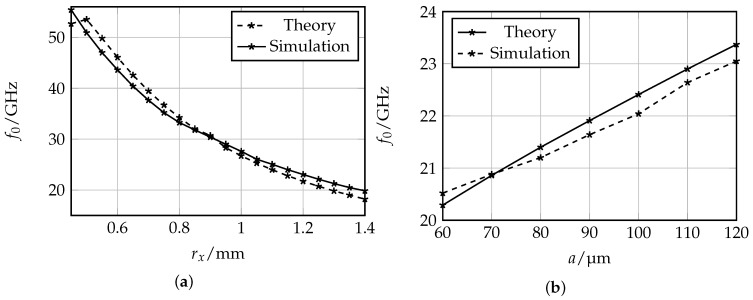
Comparison of theoretical and simulated values for the resonance frequency of oval SRRs (**a,b**). The distance between the rings is *s* = 60 μm and the semi-minor axis is $r_{y}$ = 0.45 mm.

In order to verify the accuracy of the introduced derivation for the resonance frequency of the oval SRR, a comparison between the theoretical determined and simulated resonance frequencies is shown in Figure 2. Full wave simulations of SRRs surrounded by air and excited by a plane electromagnetic wave with magnetic components in *z*-direction and electric field components in *x*-direction were performed in CST Microwave Studio with varying values for the semi-major axis $r_{x}$ and the ring width *a*. The semi-minor axis $r_{y}$ of the SRRs is set to 0.45 mm. The value corresponds to the maximum radius in *y*-direction assuming that a non-intersecting ring pair is coupled to a coplanar waveguide (CPW) transmission line and does not exceed the given size limitation of 2 mm. From the resulting plots in Figure 2, the theoretical and simulated results being in good agreement can be observed. Hence, the proposed approach to compute the resonance frequency of oval SRR by considering the geometrical relation of an ellipse can be used for further investigations in this work.

Regarding the dependencies caused by parameter variation of the ring width *a* and the semi-major axis $r_{x}$ of the oval SRR, shown in Figure 2, reasonable relations can be observed. For larger circumference of the ellipse, the total inductance $L_{\mathrm{SRR}}$ and the capacitance $C_{\mathrm{SRR}}$ increase, which leads to a decrease of resonance frequency. By increasing the ring width *a*, the inductance $L_{\mathrm{SRR}}$ is decreased, leading to higher resonance frequencies of the oval SRR.

### 2.2. Sensitivity Analysis

The main challenge regarding tumor detection is to determine relatively small permittivity changes due to abnormalities in tissue. In previous studies, the dielectric properties of ex vivo organic tissue were determined [10]. Exemplary for liver tissue, a clinical study to measure the dielectric properties of normal, malignant and cirrhotic liver tissue was carried out in 2007 [11], which came to the conclusion that the permittivity of ex vivo malignant liver tissue is 19% to 30% larger than that of normal tissue. The detection of such differences leads to high demands for the sensitivity of the sensor device. For the evaluation of the sensing mechanism, full wave simulations with CST Microwave Studio were performed when the sensor device consisting of oval SRR coupled to a CPW is loaded with material mimicking normal and malignant liver tissue, respectively. The corresponding material models are based on the dielectric properties for normal and malignant liver tissue given in [11].

**Figure 3:**
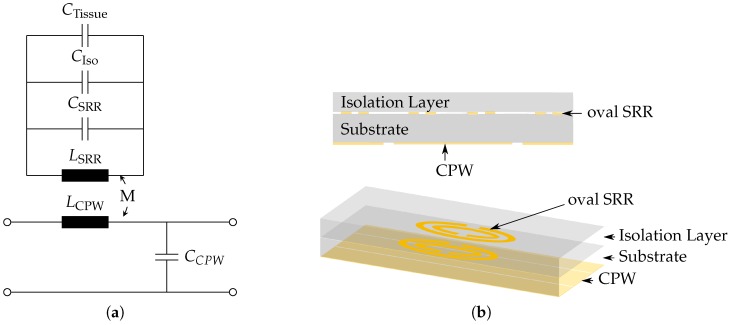
Schematic drawings of sensor with protective isolation layer. (**a**) equivalent circuit model; (**b**) cross section and perspective view.

Generally, resonant methods for sensing mechanisms have higher accuracies and sensitivities than non resonant methods [12]. However, they are more sensitive against the high losses inherent to the tissue. Therefore, an isolation layer on top of the SRRs is needed. The equivalent circuit model of SRRs coupled to a CPW and loaded with an isolation layer and tissue sample is shown in Figure 3a, and the corresponding schematic drawings of the sensor is shown in Figure 3b. The resonance frequency of this model is extended by the addition of the capacitances caused by the isolation layer $C_{\mathrm{Iso}}$ and the tissue $C_{\mathrm{Tissue}},$ and can be written as

(9) $$f_{0}=\frac{1}{2\pi }\;\cdot \;\sqrt{\frac{1}{L_{\mathrm{SRR}}\left(C_{\mathrm{SRR}}+C_{\mathrm{Iso}}+C_{\mathrm{Tissue}}\right)}}.$$

When loading the sensor with different tissue types, in this case with healthy and malignant tissue, the permittivity change of normal vs. malignant tissue leads to a decreased value for the capacitance $C_{\mathrm{Tissue}}$ while the other parameters are not influenced. The relative frequency shift can be written as

(10) $$\frac{\Delta f^{2}}{f_{0}^{2}}=\frac{f_{0,H}^{2}-f_{0,M}^{2}}{f_{0,M}^{2}}=\frac{C_{M}-C_{H}}{C_{H}+C_{\mathrm{SRR}}+C_{\mathrm{Iso}}},$$

where $C_{M}$ is the equivalent capacitance when the sensor is loaded with malignant tissue and $C_{H}$ for healthy tissue. From the relation in Equation (10) can be extracted that the main design parameters to maximize the relative frequency shift are the equivalent capacitances $C_{\mathrm{SRR}}$ and $C_{\mathrm{Iso}}$. With the limitation that the overall resonance frequency of the sensor should not exceed 12 GHz, the capacitance $C_{\mathrm{SRR}}$ can be mainly adjusted by the semi-major axis radius $r_{x}$ with values above 1 mm. In order to influence $C_{\mathrm{Iso}}$, the selected material and thickness of the isolation layer $t_{\mathrm{Iso}}$ are possible parameters to alter. For the evaluation of corresponding simulation results, a Figure of Merit (FoM) is defined that takes into account the relative frequency shift $\Delta f/f_{0}$ and the sharpness of the resonance peak presented by the normalized 1-dB bandwidth $B_{1\mathrm{dB}}/f_{0}$:

(11) $$\mathrm{FoM}=\frac{\Delta f}{B_{1\mathrm{dB}}}.$$

For the substrate and isolation layer, thin glass was selected due to its biocompatibility and proper sterilization properties. Additionally, glass provides a small permittivity value of $\varepsilon_{r,\mathrm{Glass}}=4.6$ and relatively low losses in the X-band, tanδ=0.006 at 12 GHz, to reduce the influence of $C_{\mathrm{SRR}}$ and $C_{\mathrm{Iso}}$ on the resonance frequency of the sensor. The proposed thin glass is produced with certain thicknesses, whereby the relevant values for our purposes range from 200 μm to 400 μm.

In Table 1, the trade off between the ellipse radius, the isolation layer thickness and sensitivity can be clearly identified. For a larger isolation layer, the capacitance $C_{\mathrm{Iso}}$ decreases; however, the electromagnetic field penetrates less into the targeted tissue. Regarding the semi-major axis of the oval SRR, a larger size leads to an increased capacitance $C_{\mathrm{SRR}}$ and decreased resonance frequency that results in a larger value for the normalized resonance shift. According to these results, the maximum value for the FoM was achieved for an isolation of thickness $t_{\mathrm{Iso}}$ = 300 μm and a semi-major radius of $r_{x}$ = 1.2 mm.

### 2.3. Heat Generation

**Figure 4:**
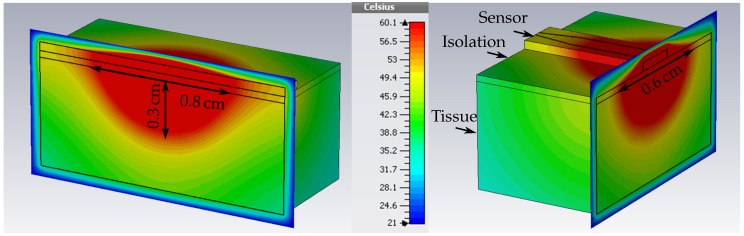
Simulated ablation zone with dimension 0.8 cm × 0.6 cm × 0.3 cm. The temperature is given in ${}^{\circ }C$.

In the treatment mode of the sensor, microwave ablation is performed. With the information about relative dielectric property changes detected by the sensing mechanism at the tip of a needle-like surgery tool, the precise position of the tumor can be defined and subsequently be ablated. For this, the input power is amplified at the specific resonance frequency of the SRR that detected abnormalities. At microwave frequencies, heat is generated due to molecules such as water in tissues that realign themselves to the applied field. The resulting kinetic energy is converted into heat. A temperature increase at the location of the tumor of up to 60 ${}^{\circ }C$ leads to irreversible damage of the cells [2]. In ablation theory, it is important to find a way to quantify electrical fields and their influence in organic tissue, especially the heat transfer that becomes more complex due to the presence of continuous blood flow through a complex network of branching vessels as well as several further biomechnanics in cells and organs. In addition, the heat effect of ablation does not only depend on the temperature increase but also on the duration of the thermal exposure. The bioheat equation, firstly introduced by Pennes [13], describes a model for the heat transfer in tissue that includes the effects of metabolism and blood perfusion. The equation is given by

(12) $$\rho c\frac{\delta T}{\delta t}=\Delta \;\cdot \;k\Delta T+{\left(\rho c\right)}_{b}\omega_{b}\left(T_{b}-T\right)+{\dot{Q}}_{met},$$

with mass density *ρ*, the specific thermal capacity *c*, the thermal conductivity *k*, and the Temperature *T* of tissue, respectively. The subscript *b* denotes these properties for blood and $\omega_{b}$ is the blood perfusion in units of volume of blood flowing per unit time per unit of tissue volume. The metabolic rate is given by ${\dot{Q}}_{met}$ [14]. In the thermal simulations of the proposed theranostic device, the corresponding ablation zone can be visualized. For that, CST Microwave Studio provides materials that take into account the proposed bioheat model. For liver tissue, the thermal parameters are the thermal capacity per volume ρc/V = 3.6 kJ·K${}^{-1}$·kg${}^{-1}$, thermal conductivity *k* = 0.469 WK${}^{-1}$·m${}^{-1}$, the blood perfusion $\omega_{b}$ = 68,000 W·K${}^{-1}$·m${}^{-3}$, and the metabolic rate ${\dot{Q}}_{\mathrm{met}}$ = 12,000 Wm${}^{-3}$. In Figure 4, the resulting temperature distribution in liver tissue is presented, when the sensor is excited at a discrete frequency with an input power of 2.1 W. The blood temperature was set to 37 ${}^{\circ }C$ and the environment temperature to 21 ${}^{\circ }C$, the standard temperature in the measurement lab. The area inside the tissue with a temperature rise up to 60 ${}^{\circ }C$ can be measured and equals 0.8 cm × 0.6 cm × 0.3 cm.

## 3. Experimental Setup

**Figure 5:**
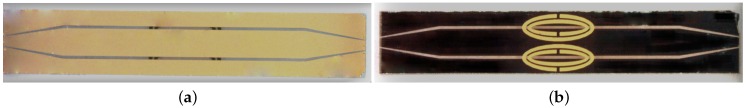
Prototype with the following dimensions: length = 12 mm, width = 2 mm, semi-major axis $r_{x}$ = 1.2 mm, semi-minor axis $r_{z}$ = 0.45 mm, ring width *a* = 0.12 mm, distance between rings *s* = 0.06 mm, split size *x* = 0.1 mm. (**a**) frontside is structured with CPW line; (**b**) backside is structured with oval SRRs.

For the evaluation of the sensing and heating functionality of the proposed design, the structures were fabricated in a photolithographic process. The resulting prototype and relevant dimensions are given in Figure 5. The lower surface of the structure is colored black due to chrome as adhesive agent for the gold metallization. In the following, the fabrication process is described more detailed and the measurement setups for the detection and treatment mode evaluation are presented.

### 3.1. Fabrication

For the fabrication of the sensor, both sides of the substrate, the front and back side, were structured. Therefore, a successive process was developed that includes the continuous protection of the not-processed side with a layer consisting of photoresist and an adhesive foil. In addition, a precise alignment is needed to ensure the accurate position of the SRR with the center point in the center of the gaps of the CPW in order to achieve the highest coupling between line and SRR. As substrate material, thin glass of height 400 μm with a permittivity of $\varepsilon_{r}$= 4.58 is used. A seed layer out of chrome and gold is vapored on each side followed by electroplating to heighten the gold metallization layer up to 2 μm.

The main objective of the design is to obtain an operating frequency below 12 GHz and simultaneously meet the critical size requirement that limits the width of the sensor to 2 mm. Therefore, the strip width of the CPW is constructed in a way offering sufficient space for the pair of SRRs to achieve a maximum possible radius in the *y*-direction without any intersections of the rings. The gap width of the CPW is adapted to obtain a 50 Ω line impedance. Towards the edges of the CPW, a linear taper is designed to enable measurements with on-wafer probes for the connection of the sensor to external equipment.

### 3.2. Measurement Setup

**Figure 6:**
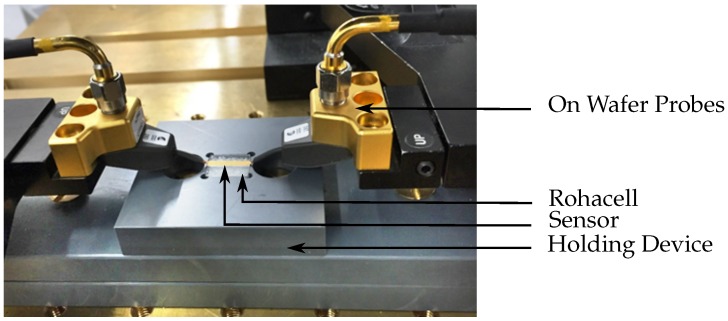
Sensor device is contacted with on wafer probes for the connection to external measurement equipment.

On wafer probes with a pitch distance of 250 μm are used to connect the CPW of the sensor structure to the specific measurement equipment that varies for the detection and treatment mode. Since the SRRs are located on the back side of the structure, a holding device was designed that provides space for the isolation layer and loading of the SRRs. In Figure 6, the measurement setup is shown when the sensor is loaded with Rohacell that has an $\varepsilon_{r}\approx 1$ to imitate the unloaded case.

#### 3.2.1. Detection Mode

**Figure 7:**
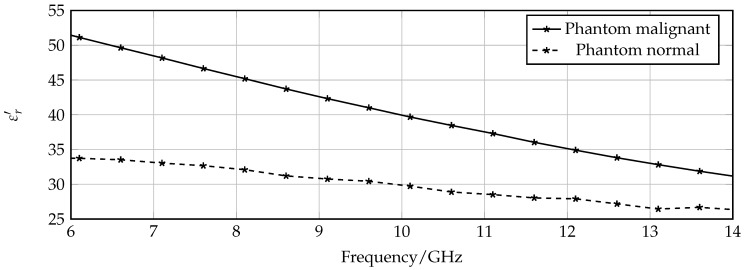
Permittvity of phantoms mimicking normal and malignant tissue.

First, the measurement setup for the detection mode is prepared. The probes are connected to a vectorial network analyzer followed by a careful calibration in the frequency range from 5 GHz to 18 GHz with 6001 measurement points, resulting in a resolution of 2.2 MHz. In order to evaluate the sensing properties, the container in the holing device is successively filled with phantoms that mimic dielectric properties of healthy and malignant tissue instead of Rohacell, and the transmission characteristics are measured. The phantoms are fabricated according to the recipe given in [15]. By changing the ratio of the main ingredients—water and oil—the dielectric properties of the phantoms can be controlled to approximate the wide band properties of ex vivo healthy and malignant liver tissues that are examined in [11]. The corresponding permittivity of the normal and malignant phantoms are given in Figure 7 for the X-band frequency range.

#### 3.2.2. Treatment Mode

For the validation of the treatment mode of the sensor, the measurement setup is changed to provide a high input power at one distinct frequency. For that, the vectorial network analyzer is replaced by a discrete source generating a signal with certain frequency and a power amplifier suitable for frequencies up to 12 GHz. The measurement setup for thermal ablation experiments was introduced in more detail in [16].

In this work, the treatment efficiency was firstly validated by ablating ex vivo beef liver tissue. The tissue is placed in the container of the holding device and covered with the isolation layer. In a first step, the resonance frequency of the SRR loaded with the liver tissue was determined using the measurement setup of the detection mode. Then, the input power is successively increased from 30 mW to 2.1 W. After three minutes, the experiment is completed and the size of the ablated tissue can be obtained by analyzing the color of the tissue.

## 4. Measurement Results

Measurements were performed for the detection mode and the treatment mode with previously introduced experimental setups. In the following, the corresponding results are presented and compared with full wave simulations.

### 4.1. Detection Mode

**Figure 8:**
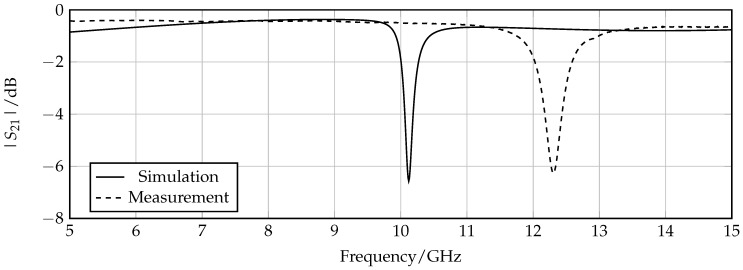
Comparison of simulated and measured transmission coefficient for the unloaded sensor.

To prove the concept of the sensing properties, measurements were performed using the proposed measurement setup for the detection mode. The first test was to check if the actual prototype shows an expected behavior by comparing the transmission coefficient measurements for an unloaded sensor with corresponding simulation results. The resulting plot is shown in Figure 8. A frequency offset of about 2 GHz between the measurements and simulation can be extracted for each measurement. One possible reason for this discrepancy can be the presence of air gaps between the sensor structure and the isolation layer that leads to an increase of resonance frequency due to the decrease of the effective surrounding permittivity in the area of the SRRs. Besides this offset, the measurement shows an expected behavior in terms of shape and depth of the resonance peak.

**Figure 9:**
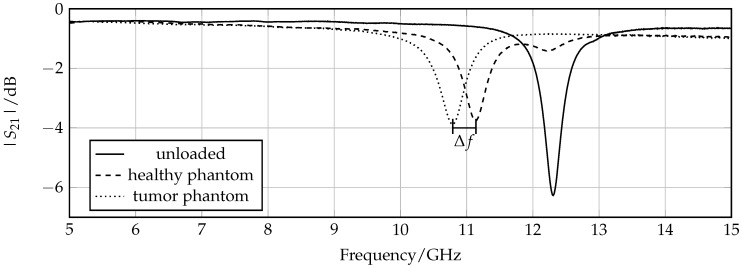
Measured transmission coefficient of sensor loaded with phantom mimicking normal and tumor tissue. The absolute frequency shift Δ*f* = 350 MHz.

The next step is to observe if the resonance frequency changes when the sensor is loaded with a phantom mimicking normal tissue compared to that mimicking tumorous tissue. The measured graphs of the two transmission coefficients in comparison with the unloaded case are presented in Figure 9. A clear resonance shift of 350 MHz that corresponds to a relative frequency shift of 3.2% is detected. Regarding the previously introduced sensitiviy analysis, the 1 dB bandwidth $B_{1\mathrm{dB}}$ = 330 MHz, which results in an FoM of 1.06. The frequency shifts, and, consequently, the FoM observed in the measurements is increased compared to simulations due to the fact that simulated material models have a smaller difference between permittivity of normal and malignant tissue. According to Figure 7, the corresponding permittivity change of the different phantoms at around 11 GHz is $\Delta \varepsilon =\varepsilon_{\mathrm{healthy}}$−$\varepsilon_{\mathrm{tumor}}\approx$ 10, and for the simulated material models $\Delta \varepsilon =\varepsilon_{\mathrm{sim},\mathrm{healthy}}$−$\varepsilon_{\mathrm{sim},\mathrm{tumor}}\approx$ 7. A further observation is that the resonance peaks of the loaded sensor are degraded compared to the unloaded case due to the losses of the phantoms mimicking organic tissue.

### 4.2. Treatment Mode

**Figure 10:**
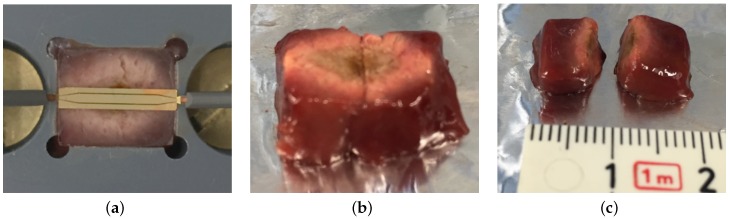
Liver tissue after thermal ablation. The ablation zone can be extracted by evaluating the color of the tissue. (**a**) ablated liver tissue in the container of the holding device; (**b**) front side of ablated liver tissue; (**c**) cross section of liver tissue to extract penetration depth.

**Figure 11:**
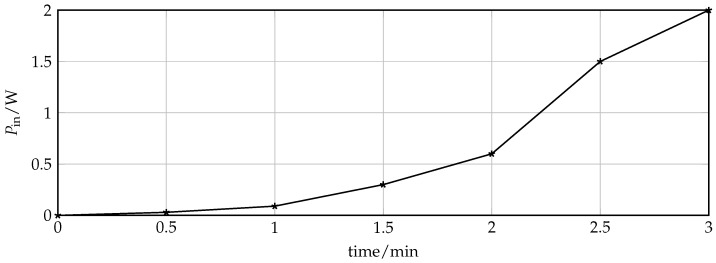
Input Power at device under test over exposure time during ablation process.

The treatment mode validation of the theranostic device is focused on the achievable dimension of the ablation zone. For this purpose, ex vivo beef liver tissue was ablated by increasing the input power at specific resonance frequency that was measured prior to the ablation procedure and equals $f_{0}$ = 11.48 GHz. Figure 10 shows the liver tissue after three minutes of exposure with successive increase of input power up to 2.1 W. The progress of input power at the device under test and time is depicted in Figure 11. Every half a minute, the input power of the discrete signal generated was increased logarithmically from 0.01 mW to 3.2 mW and amplified. The actual input power at the device under is determined with the help of a connected power meter. The initial temperature of the liver sample was 21 ${}^{\circ }C$.

The ablation zone was determined by measuring the area where the tissue is discolored from dark red towards rose. The liver sample was cut to extract the ablation depth. The resulting size equals 1.2 cm × 1 cm × 0.3 cm. Compared to the thermal simulations that were presented in Figure 10, the actual measured lesion zone has a slightly larger dimension but with a similar shape.The simulated tissue material models present averaged thermal and dielectric properties that changes even in the same tissue for instance dependent on the presence of larger blood vessels. All in all, the thermal simulation results can be seen as a good indicator of how well the theranostic tool functions and can be used for future sensor designs.

## 5. Discussion

The proposed theranostic microwave device with oval SRRs provides two modes of operation for the detection and treatment of cancerous tissue. In the following, the results concerning the introduced theoretical model of the oval SRRs as well as the detection and treatment mode properties are discussed.

### 5.1. Design

The main challenge concerning the design of a minimally invasive applicator is given by the size limitation that the sensor can be integrated in a needle-like operation tool. The general functional principle was previously proven by using circular SRR with a larger dimension in the range of several cm [17]. By simply decreasing the size of the SRR, the operating frequency increases significantly. Therefore, the evolution towards oval shaped SRRs was investigated that provides an appropriate resonance frequency and fulfills the given size requirements. The reason for aiming for a lowest possible operating frequency is to enable cost efficient, robust, and user friendly measurement setups that can be installed in a clinical environment. The theoretical model that includes the geometrical relation between circle and ellipses explains the frequency behavior of oval SRRs. It not only validates the designed structures but is also suitable for further investigations of similar resonant particles.

### 5.2. Detection Properties

The detection mode of the theranostic applicator provides a control system for thermal ablation systems to monitor the progress of the therapy almost in real-time. Regarding the simulation and experimental results of the detection mode, relative permittivity changes can be extracted to discern between healthy and malignant tissue not only prior to the ablation process but also afterwards without removing the applicator from the patient. This feature is useful to control the progress of the treatment. However, more information about in vivo dielectric properties and differences between healthy, tumorous and ablated tissue is needed for further adjustments to the sensitivity.

### 5.3. Treatment Properties

From the experimental validation of the treatment mode of the sensor, the power efficiency can be observed. In comparison with commercial microwave ablation systems that work with an input power up to 200 W, the proposed applicator requires about 2 W of input power, and, therefore, has no need of a cooling mechanism. Additionally, the size of the ablation zone obtained in the measurements is in the range of 1 cm${}^{3}$ due to conductive and forced convective heat transfer in tissue. Generally, a reduction of power consumption is expected since the proposed applicator has a higher operating frequency than commercial microwave ablation tools working at 915 MHz and 2.45 GHz. With increasing frequency, the conductivity of tissue increases, leading to a higher heat generation. Considering the ablation zone size of the proposed device, the commercial competitors are able to ablate much larger areas of up to 3 cm${}^{3}$. However, the theranostic device provides a more targeted way to focus the EM field around the resonator gaps [18]. Together with the fact that the physical size of the sensor is small, the advantage of a more controllable ablation zone can be obtained, and therefore, less healthy tissue is damaged during the thermal treatment.

## 6. Conclusions

In this work, a planar microwave sensor for the theranostic therapy of organic tissue was presented that proposes a microwave ablation applicator with a feature to detect malignant tissue before and after the therapy. The key elements of such a device with an adequate size for the integration in a needle-like operation tool are oval SRRs that work in a frequency range between 8 GHz and 12 GHz. The resonator based approach have beneficial properties to detect small relative permittivity changes due to malignancies in organic tissue. When the sensor was loaded with phantoms mimicking healthy compared to tumorous tissue, a frequency shift of 350 MHz was measured. Moreover, thermal ablation therapy can be performed by increasing the input power at the specific resonance frequency to 2.1 W. An ablation zone of 1.2 cm × 1 cm × 0.3 cm was determined for thermal measurements with a sample of ex vivo beef liver. Future work will focus on the packaging of the sensor tool and the possibility of MRI compatibility.

## Figures and Tables

**Table 1:** Sensitivity observations for different isolation layer thicknesses and semi-major axis values.

$t_{\mathrm{Iso}}$/μm	$r_{x}$/mm	$f_{0,M}$/GHz	Δf/MHz	$B_{1\mathrm{dB}}/$MHz	FoM/%
200	1	11.69	24	745	3.22
	1.2	9.77	22.1	443.2	4.98
	1.4	8.38	18	292.3	6.16
300	1	11.83	20	430	4.65
	1.2	9.88	19	269.7	7.04
	1.4	8.47	8	181	4.41
400	1	11.9	6	133	4.51
	1.2	9.95	6	183.8	3.26
	1.4	8.53	6	257.4	2.33
